# Prevention and long-term outcomes of naturally occurring canine heartworm infection in primary care settings

**DOI:** 10.3389/fvets.2023.1334497

**Published:** 2024-01-15

**Authors:** Kennedy Mwacalimba, Jo Ann Morrison, Patrick Ly, Nathaniel Spofford, Mike Yang, Emi Saito, Jenifer Sheehy, Christopher Adolph, Barbara Poulsen Nautrup, Christopher Brennan

**Affiliations:** ^1^Outcomes Research, Zoetis, Parsippany, NJ, United States; ^2^Banfield Pet Hospital, Vancouver, WA, United States; ^3^Veterinary Professional Services, Zoetis, Parsippany, NJ, United States; ^4^EAH-Consulting, Aachen, Germany; ^5^Covetrus, Portland, ME, United States

**Keywords:** dirofilaria immitis, heartworm disease, dog, prevention, outcomes

## Abstract

**Background:**

This study had two objectives: first, to examine the association between the history of heartworm preventive purchase compliance and the risk of positive heartworm tests, and second to preliminarily investigate the long-term cardiac outcomes of heartworm disease in dogs that had undergone successful adulticidal therapy.

**Methods:**

A retrospective cohort study design was used for both analyses, using anonymous transaction data from Covetrus (retrospective analysis 1) and anonymized medical records from Banfield Pet Hospital (retrospective analysis 2), both including canine patients across the USA. The first analysis examined the relative risk (RR) of a positive heartworm test in dogs with lapses in heartworm preventive purchase history compared to dogs that had no history of a preventive purchase six to 24 months prior to the test. In the second analysis, a long-term evaluation of structured diagnostic codes pertaining to cardiac diseases and risk assessment of outcomes was performed in dogs that had previously been successfully treated for heartworm disease compared to dogs that never had a positive heartworm test.

**Results:**

83,478 unique patients were included in the first analysis. Compared to 32,413 dogs with no history of a heartworm preventive purchase, 44,410 dogs with lapses in monthly preventive purchases had a reduced risk of testing positive for heartworm disease (RR = 0.36, *p* < 0.0001). Dogs (*n* = 6,655) with lapses in injectable heartworm preventive administration had a decreased risk of a positive test versus dogs with no preventive purchases (RR = 0.15, *p* < 0.0001), as well as versus dogs with lapses in monthly heartworm preventive purchases (RR = 0.28, *p* = 0.0024). In the second analysis, 6,138 patients treated for heartworm infection were found to have significantly (*p* < 0.001) elevated risks of right heart failure (RR = 3.59), left heart failure (RR = 1.83), or cardiomyopathy (RR = 2.79) compared to 4,022,752 patients that never had a positive heartworm test.

**Conclusion:**

This study highlights the importance of compliance with heartworm preventive guidelines, to reduce the risk of heartworm disease in dogs, which is not only a potentially life-threatening condition in the short-term but also associated with long-term negative cardiac outcomes.

## Background

The potentially life-threatening canine heartworm disease, caused by the nematode *Dirofilaria immitis*, continues to be diagnosed in the USA, with 1.15% of heartworm tests being positive in 2022 (1). Diagnosis of heartworm infection in clinical practice relies upon detecting antigen of *D. immitis* and microfilaria testing (2, 3). Additional test methods, such as radiography and echocardiography, are also useful for confirming the diagnosis and staging the severity of heartworm disease (3, 4). While most dogs identified through screening tests are asymptomatic, many have lesions of the pulmonary arterial tree, the lungs, and secondarily the heart, which can lead to irreversible pulmonary hypertension and right-sided heart failure. The severity of the disease is correlated to worm burden and conversely, the size of the host (5), exercise activity, duration of infection and development of complications such as eosinophilic pneumonitis (4). The arsenical drug, melarsomine dihydrochloride is the only adulticidal ingredient approved by the FDA (3, 6). Following melarsomine administration thromboembolic events are predicted and strict exercise restriction is mandatory to decrease the severity of sequelae. Failure to restrict the exercise of the dog can worsen the effects of thromboembolic events, resulting in pulmonary hypertension and heart failure (4).

In order to maintain animal health and welfare, the mainstay of heartworm management is to prevent the development of adult worms, which are responsible for heartworm disease. This preventive approach relies on a single drug class, the macrocyclic lactones, which kill the extremely sensitive third and fourth stage larvae of *D. immitis* (5). In the USA, there are three common forms of macrocyclic lactones available: monthly oral tablets/chews, monthly topical liquids, and injectable extended-release moxidectin suspension (7). The American Heartworm Society (AHS) recommends year-round heartworm protection for all dogs in the USA. Even though continuous, year-round transmission may not occur throughout the USA, the administration of macrocyclic lactones over 12 months likely enhances compliance (3) and repeated administrations also increase the effectiveness of commonly prescribed monthly heartworm preventives and is part of the label recommendation of some macrocyclic lactones (8). There are both individual and population-level benefits to year-round protection, namely the prevention of infection of individual animals and the reduction of the overall prevalence of adult and reproducing *D. immitis* in domestic dogs (3). Even a single missed or delayed monthly preventive dose can lead to heartworm disease (3). The monthly heartworm preventives do not display prospective or residual efficacy against *D. immitis*, but rather have a “reach-back” efficacy against past inoculations (9), which is 30 days (10).

Although resistance against macrocyclic lactones has been confirmed in some strains of *D. immitis* in the Lower Mississippi River Valley (LMRV) (9, 11), non-compliance is the main contributor for heartworm disease in dogs (12). Approximately only a third of all medicalized dogs in the USA receive one or more doses of heartworm preventive annually (13). In dogs receiving heartworm preventives, year-round compliance is low, as reported in a retrospective analysis of transaction data from 4,615 clinics and 3.5 million dogs throughout the USA, evaluating the purchase compliance with different heartworm preventives. The average annual number of monthly heartworm preventives (oral and topical products) was 7.3, i.e., dogs were protected from heartworm disease 7.3 of 12 months only. Longer periods of protection were on average achieved with the injectable extended-release moxidectin formulations, being calculated to 8.1 months (ProHeart^®^ 6, which provides 6 months of protection with one injection) and 12 months (ProHeart^®^ 12, which provides 12 months of protection with one injection) (14).

The primary objective of the current study was to understand the association between heartworm preventive purchase compliance and the risk of positive heartworm tests in dogs seen in general veterinary practice in the USA. The secondary objective was to investigate the hypothesis that dogs tested positive for heartworm disease and subsequently successfully treated still had increased risks for negative cardiac outcomes compared to dogs that never had a positive test recorded within primary care hospitals in the USA.

## Methods

Two retrospective analyses were conducted, using transaction data (retrospective analysis 1) and electronic medical records (retrospective analysis 2). Both analyses followed the guidelines and checklist for a systematic approach to compliance and persistence studies using retrospective databases (15).

### Retrospective analysis 1

To understand the association between heartworm preventive purchase compliance and the risk of positive heartworm tests in dogs, transaction data were used from a centralized database of more than 6,000 practices across all states of the USA, managed by Vetstreet Veterinary Practice Management Services (Covetrus Inc., Portland, ME, United States). Covetrus provided anonymized transaction data from practices that met the pre-defined inclusion criteria. Practices were included which had at least 90% of their heartworm test results recorded (positive or negative), and all practices had to have both positive and negative results documented during the observation period. From these practices, dogs were identified which were subject to a heartworm disease test between November 2019 and October 2020. These patients were divided into 3 cohorts according to their heartworm preventive purchases within six and 24 months prior to the test: (A) dogs with transactions of any monthly heartworm preventive, with all monthly heartworm preventives (oral and topical products) being considered one preventive modality; (B) dogs with a historic purchase of injectable extended-release moxidectin formulation (ProHeart^®^6 or ProHeart^®^12); and (C) dogs with no history of a heartworm product transaction. In cohorts A and B, only lapsed patients were included, i.e., patients which had gaps of at least 30 days in monthly heartworm preventive product coverage or missing or postponed re-injections of the extended-release moxidectin formulations based on their purchase history during the look-back period. Purchases made within 6 months prior to the heartworm test were not considered, as dogs tested positive were likely exposed to the heartworm larvae six or more months prior to diagnosis. Accordingly, the look-back period for purchases of heartworm preventive medication over all dogs was December 2017 until September 2020. Dogs had to be still active within a practice for at least 12 months prior to the heartworm test, to ensure that the patients were current, i.e., could have purchased heartworm preventives in the respective clinic if wanted.

Finally, dogs in the three cohorts were classified according to their test result (positive or negative). Dogs tested both positive and negative during the same visit were excluded, implying either test inaccuracy, potential retest, or clerical error. Dogs with no record of test result were also excluded.

It was assumed that all preventives were 100% effective against *D. immitis* and all heartworm positive tests were due to gaps in protection. The percentages of dogs tested positive were calculated for all dogs and within the three cohorts. The relative risk (RR) of having a positive heartworm disease test result was estimated between the cohorts. All calculations including the corresponding *p*-values (at alpha level of 0.05) were performed according to published standards (16).

### Retrospective analysis 2

This retrospective study used electronic medical records of Banfield Pet Hospitals. During the study period there were over 1,000 hospitals located in 42 U.S. states, the District of Columbia and Puerto Rico linked by the same proprietary pet medical record system (PetWare^®^). The clinic system contained structured and unstructured fields. Structured fields included pet demographics, physical exam observations, lab findings, clinical signs, diagnostic and ailment codes, as well as invoice information on services provided and products dispensed. Unstructured fields allowed clinicians to enter free-text medical notes. Data were uploaded nightly from all clinics to a centralized data warehouse, where it was available for analysis.

For the analysis of long-term cardiac pathology after heartworm disease the following outcomes of interest were defined: primary outcome of interest was right-sided heart failure, being a potential long-term consequence after heartworm disease (17). It corresponded to the clinical diagnosis “heart failure, right sided” and/or ascites in the software. The outcomes of interest also included left-sided heart failure, which consisted of the clinical diagnosis “heart failure, left sided” and/or pulmonary edema, as well as cardiomyopathy, which consisted of the clinical diagnosis “cardiomyopathy, dilated, congestive” and “cardiomyopathy, canine dilated.”

For the analysis of a potential correlation between the cardiac pathologies and previous heartworm disease dogs were included that had at least one heartworm test conducted at any in-network hospital between January 31, 2005 and December 27, 2014, thereby providing time to allow for the longitudinal analysis. The study population was classified into two cohorts: a non-exposed group and an exposed group. The non-exposed group included dogs that never had a positive heartworm test or diagnosis of heartworm disease during the study period and were not previously diagnosed with any of the cardiac outcomes of interest prior to their first negative heartworm test. Dogs were included in the exposed group according to the following in−/exclusion criteria:

- Dogs must have had a positive heartworm antigen test or be diagnosed with heartworm disease for the first time during the 10 years period.
- Heartworm test positive dogs received treatment with the arsenic-based adulticide melarsomine dihydrochloride. Dogs that received alternative therapy, such as “slow kill” therapy (18), which is not recommended by the AHS (3), were excluded from analysis. In addition the decision was made to only include dogs that received adulticide therapy in an attempt to keep the comparison analysis as straight forward as possible.
- Dogs must have tested negative on heartworm antigen test 6–12 months after administration of the last arsenic-based treatment.
- Dogs previously diagnosed with any of the cardiac outcomes of interest prior to the first heartworm-positive test or disease diagnosis were excluded.
- Dogs that were subsequently tested positive on heartworm antigen after previously testing negative post melarsomine treatment were excluded, to evaluate long term outcomes of dogs in which parasite clearance was clearly established.

Both cohorts were followed until the end of observation period (September 2022) and any outcome of interest (right-sided heart failure, left-sided heart failure, or cardiomyopathy) was recorded.

Data extracted from the records included state of primary hospital, birth date, breed, pet first visit date, study entry date, pet last visit date, pet status (e.g., active, euthanized), number of arsenic-based adulticide treatments administered and diagnosis of outcomes of interest and age at diagnosis. Statistical analysis was performed with Microsoft Excel^™^. Risk ratios between exposed and non-exposed dogs were calculated for each outcome, with chi-square test performed to evaluate statistical significance at alpha level of 0.05 (16).

## Results

### Retrospective analysis 1

The original sample included 4,711 practices, from which 4,574 practices had conducted at least 1 heartworm disease test. One hundred twenty-eight practices fulfilled the required number of ≥90% test results recorded and 50 practices had positive and negative results recorded for their patients, representing the practice subset. From these practices 83,478 unique patients were included in the analysis. Of all dogs, 38.8% had no heartworm preventive purchase history (cohort C), 53.2% had purchase histories of monthly heartworm preventives (cohort A), and 8.0% had received an extended-release moxidectin formulation during the look-back period (cohort B). The percentage of dogs tested positive over all dogs was 0.53%. The percentages of dogs tested positive in the three cohorts A, B, and C were 0.32, 0.14, and 0.90%, respectively (Table 1).

**Table 1:** Outcome of heartworm tests depending on the purchase of heartworm (HW) preventives in dogs.

Cohort	Number of dogs tested	Number of dogs tested positive	Percentage of dogs tested positive (%)	RR for positive test (compared to no HW preventive)	*p*-value for RR
All dogs	83,478	444	0.53	N.A.	N.A.
HW preventive modality					
Monthly HW preventive	44,410	142	0.32	0.36	<0.0001
Injectable moxidectin formulation	6,655	9	0.14	0.15	<0.0001
No HW preventive	32,413	293	0.90	N.A.	N.A.

Monthly HW preventives included all oral and topical products for monthly application; injectable moxidectin formulations included ProHeart^®^6 and ProHeart^®^12. RR, relative risk.

Even with lapses in preventive purchases, dogs on any heartworm preventive medication had significantly (*p* < 0.0001) reduced risks of a positive heartworm test compared to dogs with no history of a heartworm preventive purchase. The RR of dogs on monthly heartworm preventives was 0.36 and the RR of dogs with a history of an injectable heartworm preventive purchase was 0.15. Accordingly, dogs receiving no heartworm preventive were 2.7 times more likely to be positive compared to dogs receiving monthly heartworm preventives and 6.7 times more likely to be positive compared to dogs previously prescribed an injectable heartworm preventive. When comparing cohorts A and B, the risk of a positive heartworm test was significantly (*p* = 0.0024) reduced (RR = 0.28) in dogs which received the injectable heartworm preventives compared to dogs with a purchase history of monthly heartworm preventives.

### Retrospective analysis 2

For the second objective, records were collected from over 1,000 hospitals. The total number of exposed dogs, i.e., tested positive for heartworm disease and being successfully treated, was 6,138, whereas the number of dogs never tested positive for heartworm disease (non-exposed dogs) was 4,022,752. The percentage of dogs diagnosed with any of the defined heart diseases was 2.66 and 1.21% in the exposed and non-exposed group, respectively. The incidences of right heart failure, left heart failure, and cardiomyopathy were 0.68, 1.71, and 0.26% (exposed group) and 0.19, 0.93, and 0.09% (non-exposed group), respectively. Thus, dogs that were positive for heartworm infection and successfully underwent treatment were found to have significantly (*p* < 0.0001) increased risks for right heart failure (RR = 3.59), left heart failure (RR = 1.83), and cardiomyopathy (RR = 2.79) compared to non-exposed dogs. Overall, dogs recovered from heartworm disease had a RR of 2.20 (*p* < 0.0001) compared to the unexposed group of dogs (Table 2).

**Table 2:** Number of clinical diagnoses of canine cardiac pathology depending on the presence or absence of a previous heartworm diagnosis.

Outcome of interest	Dogs successfully treated for heartworm disease		Dogs not tested positive for heartworm disease		RR	*p*-value
	Positive diagnosis/all dogs	%	Positive diagnosis/all dogs	%		
Any diagnosis	163/6,138	2.66%	48,665/4,022,752	1.21%	2.20	<0.0001
Right-sided heart failure	42/6,138	0.68%	7,639/4,022,752	0.19%	3.59	<0.0001
Left-sided heart failure	105/6,138	1.71%	37,280/4,022,752	0.93%	1.83	<0.0001
Cardiomyopathy	16/6,138	0.26%	3,746/4,022,752	0.09%	2.79	<0.0001

The relative risks (RR) of a cardiac pathology was calculated for dogs treated for heartworm disease compared to dogs previously not tested positive for heartworm disease.

The median age in the exposed cohorts for right heart failure, left heart failure, and cardiomyopathy were 10.08 years, 10.72 years, and 10.34 years, respectively. Corresponding values for the non-exposed cohort were 10.82 years, 12.02 years, and 10.75 years, respectively. Table 3 provides the percentage distribution of age groups per cohort and outcome.

**Table 3:** Distribution of the age of dogs at diagnosis of cardiac pathologies in exposed dogs (previously tested positive for heartworm disease and successfully treated) and dogs never tested positive for heartworm disease (non-exposed dogs).

Age at diagnosis	0–1 years (%)	1–2 years (%)	2–3 years (%)	3–4 years (%)	4–5 years (%)	5–6 years (%)	6–7 years (%)	7–8 years (%)	8–9 years (%)	9–10 years (%)	10–11 years (%)	11–12 years (%)	12–13 years (%)	13–14 years (%)	14–15 years (%)	15+ years (%)	Median Age (years)
Right heart failure																	
Exposed dogs	0.00	0.00	0.00	4.76	0.00	4.76	2.38	7.14	9.52	21.43	9.52	16.67	11.90	4.76	4.76	2.38	10.08
Non-exposed dogs	0.49	1.10	1.19	1.70	2.07	2.98	4.56	5.56	8.68	10.96	12.87	13.98	12.60	10.31	6.16	4.77	10.82
Left heart failure																	
Exposed dogs	0.00	0.00	0.00	0.96	1.92	2.88	1.92	8.65	8.65	14.42	15.38	8.65	9.62	15.38	6.73	4.81	10.72
Non-exposed dogs	0.11	0.23	0.22	0.30	0.48	0.83	1.61	2.95	5.64	9.15	13.12	14.92	15.83	13.92	10.43	10.27	12.02
Cardiomyopathy																	
Exposed dogs	0.00	0.00	0.00	0.00	6.25	0.00	6.25	18.75	0.00	6.25	31.25	6.25	12.50	0.00	6.25	6.25	10.34
Non-exposed dogs	0.29	1.15	1.31	1.71	2.36	3.51	4.13	6.27	8.63	10.80	12.62	13.72	12.35	9.51	6.51	5.12	10.75

## Discussion

The objective of our study was twofold: Firstly, to examine the association between the history of heartworm preventive purchase compliance and the relative risk of a positive heartworm test, and secondly to investigate long-term outcomes in dogs that had successful adulticidal therapy after testing positive for heartworm infection.

The first analysis found that dogs even with a history of lapses in heartworm preventive purchases had a reduced risk of positive heartworm tests compared to dogs having no history of a heartworm preventive purchase six to 24 months prior to the test. Dogs recently receiving no heartworm preventive were 6.7 times more likely to be positive for heartworm disease compared to dogs with lapses in purchase of an injectable heartworm preventive, and 2.7 times more likely to be positive compared to dogs with lapses in purchase compliance with monthly heartworm preventives. Results indicate that any heartworm medication is better than no preventive approach. These results align with findings from a previous study. In an analysis of over 11 million veterinary medical records from the USA, dogs receiving a heartworm preventive were significantly less likely to develop heartworm disease compared to untreated dogs (19). However, with lapses in the history of purchase, dogs are at risk of heartworm disease, therefore the goal should be to educate the dog owner to keep their dogs year-round on heartworm preventives as recommended by the AHS (3). The risk of a positive test was reduced in dogs receiving the injectable moxidectin formulation compared to monthly heartworm preventives. As the actual number and time-periods of gaps in prevention was not recorded, it can only be assumed that the added preventive benefit with injectable moxidectin was related to increased compliance with to the longer-acting moxidectin formulations, i.e., resulting in less and/or smaller gaps compared with monthly heartworm preventives, at least in non-resistant strains. This assumption is mirrored by results reported for the purchase compliance in the USA, as the annual time dogs were protected was calculated longer in dogs receiving the injectable extended release moxidectin formulation (8.1 months with PH6 and 12 months with PH12) compared with dogs on monthly heartworm preventives (7.3 months) (14).

We do not know the tests used for heartworm diagnosis in the various clinics, as this was not consistently captured in the transaction database. Accordingly, the sensitivity and specificity of the underlying tests and the correctness of test results could not be assessed. However, if the selection of test was similar between cohorts, i.e., was not related to the presence or absence of heartworm preventive purchases, the impact of false positive and negative results would be balanced when calculating the RR between cohorts.

In our study, the incidence of a positive heartworm test over all dogs was 0.53% and therefore approximately half of the incidence (1.15%) reported for 2022 in the USA (1). Analyses over years and states reported incidences in the USA between 1.11% in 2013 and 1.28% in 2016, with a great variability of incidence per state. When excluding the southeastern states including the LMRV, where resistant strains of *D. immitis* have been proven (20), percentages of positive heartworm test were between 0.56% in 2013 and 0.62% in 2016 (13). Although practices included in our analysis were distributed over the entire US, an underrepresentation of practices in the southeastern region might be – among others – a possible explanation for the different incidences. However, with resistant *D. immitis* strains mainly occurring in the southeastern states, positive heartworm diagnoses could have been due to resistance rather than lapses in heartworm preventives in these areas. With an assumed lower representation of practices from this region in our analysis, the positive test results are more likely to be due to lapses in preventive medication rather than resistance, which was a basis hypothesis in our study.

A limitation of this study refers to the use of transaction records, which is only a proxy for compliance. With monthly heartworm preventives, purchase history may not accurately represent the number of doses successfully administered to a pet (21), nor does it capture purchases made outside the veterinary clinic (14). Therefore, potential compliance gaps identified in the transaction records of monthly heartworm medications might be filled by purchases outside of the channels examined. We believe, however, that this limitation is only of minor importance in our study. In case of no gaps in heartworm prevention, observed heartworm diseases would be due to resistant strains of *D. immitis*, which are mainly found in the LMRV, a region where included practices assumingly might be underrepresented. However, we cannot exclude the possibility of resistant strains of *D. immitis* rather than non-compliance being the reason for some of the positive heartworm test results recorded in our analysis.

The second part of our study showed that a positive heartworm test and subsequent successful adulticidal therapy in a primary care setting still leaves the dogs at increased risk of cardiac pathology over time compared with dogs never diagnosed with heartworm disease. These findings are in line with results of a study on 34 client-owned dogs which underwent adulticide treatment after positive antigen test. The dogs were followed over 120 days and at the end of the study dogs still had pulmonary hypertension, which is a risk factor for subsequent heart failure (22). In another study, there was no significant improvement in pulmonary damage 10 months after elimination of the parasites and the authors suggested that vascular changes induced by heartworm disease are chronic and may not be reversible (23).

Right-sided heart failure is a reported long-term risk in dogs after heartworm disease (17) and was also associated with the highest RR in exposed dogs in our study (RR = 3.6). We also considered left-sided heart failure and dilated cardiomyopathy to account for additional manifestations of heart pathology diagnosis. Relative risks were also higher for these two heart pathologies in exposed dogs, thus supporting the hypothesis that cardiac outcomes were worse in dogs positive for heartworm infection compared to dogs never tested positive for heartworm in a primary care setting. The absence of board-certified specialty oversight in case management represents a potential limitation in our study. However, in a primary care setting it is possible that patients are not referred as clients may have to make difficult decisions around financial investments in the care of their pet, e.g., refusing a specialist referral.

There was likely variability in the amount of disease staging across the population, with clinicians deciding the appropriate amount of staging pursued, prior to initiating adulticide therapy. This, along with presumptive variability in the amount of diagnostic workup prior to arriving at a clinical diagnosis, may be other study limitations. But this was determined to be representative of the state of clinical practice in primary care settings. Being restricted to primary care settings, our results cannot be extrapolated to the entire dog population. However, if it may be reasonably assumed that dogs under the care of boarded specialists would be more severely affected by heartworm disease, our findings of significant increases of long-term cardiac pathology would be even more compelling. In an ideal research setting, a prospective study, following dogs tested positive or negative for heartworm disease would remove many of these limitations and could also control for other patient factors, such as breeds, sizes, genetics, and others. However, the expected timeline of such a prospective study might represent a substantial barrier.

We also recorded the age of dogs at diagnosis of the outcomes of interest. Although the median age was slightly lower in dogs with positive heartworm disease history, no definite conclusions should be drawn. In the exposed group, dogs positive for the considered cardiac pathologies were 3 years of age or older. It is hypothesized that this can be explained by the time between infection and development of heartworm disease, the treatment duration, and the subsequent occurrence of heart failure. In the non-exposed group, also young dogs (age groups ≤3 years) were diagnosed with the outcomes of interest. In many of these young dogs, a congenital or hereditary heart problem can be rationally assumed. However, excluding dogs ≤3 years would further increase the median age of diagnosis in this group, increasing the difference between non-exposed and exposed groups.

Data included in our retrospective analyses were derived from two different databases, contexts, and patient populations. Outcomes from first retrospective analysis are not necessarily related to the findings in the second retrospective analysis. Nevertheless, the two retrospective analyses provide compelling, real-world evidence in areas where prospective studies are limited, due to ethical reasons (i.e., withholding heartworm preventive medication in dogs) or practical limitations (i.e., timely limitations). Additional analyses on other patient outcomes, such as survival times, need for cardiac medications, and others, could provide more evidence.

## Conclusion

This study highlights the importance of compliance with heartworm preventive guidelines, to reduce the risk of heartworm disease in dogs, causing not only increased morbidity in the short term, but also potentially life threatening long-term outcomes.

## Data availability statement

The datasets presented in this article are not readily available because they are the property of the providing companies (Banfield and Covetrus). Requests to access the datasets should be directed to KM (kennedy.mwacalimba@zoetis.com).

## Author contributions

KM: Conceptualization, Data curation, Supervision, Writing – review & editing. JAM: Conceptualization, Writing – review & editing. PL: Conceptualization, Writing – review & editing. NS: Conceptualization, Writing – review & editing. MY: Conceptualization, Writing – review & editing. ES: Conceptualization, Writing – review & editing. JS: Conceptualization, Writing – review & editing. CA: Conceptualization, Writing – review & editing. BPN: Data curation, Writing – original draft. CB: Conceptualization, Writing – review & editing.

## References

[1] Companion Animal Parasite Council. (2022). Heartworm canine, parasite prevalence maps. Available at: https://capcvet.org/maps/#/2022/all-year/heartworm-canine/dog/united-states (accessed: March 5, 2023).

[2] Little S Saleh M Wohltjen M Nagamori Y. Prime detection of Dirofilaria immitis: understanding the influence of blocked antigen on heartworm test performance. Parasit Vectors. (2018) 11:186. doi: 10.1186/s13071-018-2736-5, PMID: 29554955 PMC5859648

[3] Nelson T McCall JW Jones S Moorhead A. (2018). Current canine guidelines for the prevention, diagnosis, and Management of Heartworm (Dirofilaria immitis) infection in dogs. Available at: https://www.heartwormsociety.org/veterinary-resources/american-heartworm-society-guidelines (accessed March 05, 2023)

[4] Bowman DD Atkins CE. Heartworm biology, treatment, and control. Vet Clin North Am Small Anim Pract. (2009) 39:1127–58. doi: 10.1016/j.cvsm.2009.06.003 19932367

[5] Prichard RK. Macrocyclic lactone resistance in Dirofilaria immitis: risks for prevention of heartworm disease. Int J Parasitol. (2021) 51:1121–32. doi: 10.1016/j.ijpara.2021.08.006, PMID: 34717929

[6] Jacobson LS DiGangi BA. An accessible alternative to Melarsomine: “Moxi-doxy” for treatment of adult heartworm infection in dogs. Front Vet Sci. (2021) 8:702018. doi: 10.3389/fvets.2021.702018, PMID: 34386540 PMC8353148

[7] Noack S Harrington J Carithers DS Kaminsky R Selzer PM. Heartworm disease – overview, intervention, and industry perspective. Int J Parasitol Drugs Drug Resist. (2021) 16:65–89. doi: 10.1016/j.ijpddr.2021.03.004 34030109 PMC8163879

[8] Bowman DD. Heartworms, macrocyclic lactones, and the specter of resistance to prevention in the United States. Parasit Vectors. (2012) 5:138. doi: 10.1186/1756-3305-5-138, PMID: 22776618 PMC3406940

[9] Diakou A Prichard RK. Concern for Dirofilaria immitis and macrocyclic lactone loss of efficacy: current situation in the USA and Europe, and future scenarios. Pathogens. (2021) 10:1323. doi: 10.3390/pathogens10101323, PMID: 34684273 PMC8541013

[10] Bowman DD Drake J. Examination of the “susceptibility gap” in the treatment of canine heartworm infection. Parasit Vectors. (2017) 10:513. doi: 10.1186/s13071-017-2433-9, PMID: 29143689 PMC5688481

[11] Savadelis MD McTier TL Kryda K Maeder SJ Woods DJ. Moxidectin: heartworm disease prevention in dogs in the face of emerging macrocyclic lactone resistance. Parasit Vectors. (2022) 15:82. doi: 10.1186/s13071-021-05104-7, PMID: 35277180 PMC8915515

[12] Atkins CE Murray MJ Olavessen LJ Burton KW. Heartworm “lack of effectiveness” claims in the Mississippi delta: computerized analysis of owner compliance – 2004–2011. Vet Parasitol. (2014) 206:106–13. doi: 10.1016/j.vetpar.2014.08.013 25440944

[13] Drake J Wiseman S. Increasing incidence of Dirofilaria immitis in dogs in USA with focus on the southeast region 2013–2016. Parasit Vectors. (2018) 11:39. doi: 10.1186/s13071-018-2631-0, PMID: 29343304 PMC5773159

[14] Mwacalimba K Sears D Brennan C Poulsen Nautrup B Sheehy J. Retrospective analyses of heartworm (Dirofilaria immitis) disease and ectoparasite preventive medication compliance in veterinary practices in the USA. Parasit Vectors. (2023) 16:149. doi: 10.1186/s13071-023-05735-y 37106437 PMC10142219

[15] Peterson AM Nau DP Cramer JA Benner J Gwadry-Sridhar F. A checklist for medication compliance and persistence studies using retrospective databases. Value Health. (2007) 10:3–12. doi: 10.1111/j.1524-4733.2006.00139.x 17261111

[16] Altman DG. Practical statistics for medical research. London, UK: Chapman & Hall (1991).

[17] Atkins CE. Complications of heartworm infection. In: World Small Animal Veterinary Association World Congress Proceedings (2003). Bangkok, Thailand, October 24–27, 2003; 2003. Available at: https://www.vin.com/apputil/content/defaultadv1.aspx?pId=8768&catId=18809&id=3850100 (accessed: May 05, 2023)

[18] Moorhead A., American heartworm society. The AHS protocol vs. slow kill, (2018). Available at: https://www.heartwormsociety.org/veterinary-resources/veterinary-education/ahs-board-speaks-out/507-the-ahs-protocol-vs-slow-kill (accessed: May 07, 2023).

[19] Glickman LT Glickman NW Moore GE Lok JB McCall JW. Comparative effectiveness of sustained-release moxidectin (ProHeart 6) and ivermectin (Heartgard plus) for the prevention of heartworm infection in dogs in the United States. Intern J Appl Res Vet Med. (2006) 4:339–54.

[20] Wolstenholme AJ Evans CC Jimenez PD Moorhead AR. The emergence of macrocyclic lactone resistance in the canine heartworm, Dirofilaria immitis. Parasitology. (2015) 142:1249–59. doi: 10.1017/S003118201500061X 26040450

[21] Lavan R Heaney K Vaduvoor SR Tunceli K. A comparative analysis of heartworm medication use patterns for dogs that also receive ectoparasiticides. Parasit Vectors. (2018) 11:493. doi: 10.1186/s13071-018-3076-1 30170612 PMC6119298

[22] Serrano-Parreño B Carretón E Caro-Vadillo A Falcón-Cordón S. Pulmonary hypertension in dogs with heartworm before and after the adulticide protocol recommended by the American heartworm society. Vet Parasitol. (2017) 236:34–7. doi: 10.1016/j.vetpar.2017.02.001, PMID: 28288761

[23] Falcón-Cordón Y Montoya-Alonso A Caro-Vadillo A Matos-Rivero JI Carretón E. Persistence of pulmonary endarteritis in canine heartworm infection 10 months after the eradication of adult parasites of Dirofilaria immitis. Vet Parasitol. (2019) 273:1–4. doi: 10.1016/j.vetpar.2019.07.008, PMID: 31442886

